# Association between statin use and the prognosis of patients with acute myocardial infarction complicated with diabetes

**DOI:** 10.3389/fcvm.2022.976656

**Published:** 2022-08-08

**Authors:** Xuehao Lu, Luming Zhang, Shaojin Li, Dan He, Tao Huang, Hongsheng Lin, Haiyan Yin, Jun Lyu

**Affiliations:** ^1^Department of Intensive Care Unit, The First Affiliated Hospital of Jinan University, Guangzhou, China; ^2^Department of Orthopaedics, The First Affiliated Hospital of Jinan University, Guangzhou, China; ^3^Department of Anesthesiology, Women's and Children's Hospital of Hengyang, Hengyang, China; ^4^Department of Clinical Research, The First Affiliated Hospital of Jinan University, Guangzhou, China; ^5^Guangdong Provincial Key Laboratory of Traditional Chinese Medicine Informatization, Guangzhou, China

**Keywords:** statin, acute myocardial infarction, diabetes, lipid-lowering drugs, cardiovascular disease

## Abstract

**Background:**

Type 2 diabetes leads to an increase in the prevalence of lipid abnormalities, which increases the risk of cardiovascular disease. Therefore, current guidelines generally recommend the use of moderate or high-intensity statins in patients with type 2 diabetes. There are still few studies on the overall risk benefit balance of statins for acute myocardial infarction (AMI) patients with diabetes. Compared with other types of lipid-lowering drugs, the advantage of statins for the prognosis of patients with AMI has not yet been determined. We investigated the effects of statins and non-statins on intensive care unit (ICU) and inpatient mortality in patients with AMI and diabetes.

**Methods:**

This study retrospectively collected all patients with AMI and diabetes in the Medical Information Mart Intensive Care-IV database. We assessed ICU and in-hospital mortality rates during hospitalization in both groups. The clinical end point was in-hospital mortality and ICU mortality. Kaplan-Meier and Cox proportional-hazards regression models were applied to analyze the correlation between the two groups and the outcomes.

**Results:**

Data on 1,315 patients with AMI and diabetes were collected, among which 1,211 used statins during hospitalization. The overall in-hospital mortality of patients with AMI and diabetes was 17.2%, and the total ICU mortality was 12.6%. The in-hospital mortality was lower for the statin group than for the non-statin group (13.9% and 55.8%, respectively). Kaplan-Meier survival curves demonstrated that survival probability was higher in the statin group than in the non-statin group. In the cohort without hyperlipidemia, the statin group had lower risks of ICU death (HR = 0.12, 95% CI = 0.04–0.40) and in-hospital death (HR = 0.36, 95% CI = 0.16–0.84) compared with the non-statin group.

**Conclusions:**

Statins can significantly reduce ICU and in-hospital mortality rates in patients with AMI and diabetes. Even in the population without hyperlipidemia, statins can still reduce the mortality in patients with AMI and diabetes.

## Introduction

Increases in the incidence rates of obesity, metabolic syndrome, and diabetes have led to cardiovascular disease (CVD) becoming the most common disease leading to death and decreased quality of life, and this adverse situation may further escalate in the near future (1). Diabetes and dyslipidemia are independent risk factors related to the incidence of atherosclerotic CVD (2). The risk of death due to CVD is 3- to 6-fold higher in patients with diabetes than in those without diabetes (3). Lipid-lowering therapy for patients with diabetes is therefore an important measure for reducing the CVD risk. The UK Prospective Diabetes Study identified elevated low-density lipoprotein (LDL) cholesterol as the leading coronary risk factor in patients with diabetes (4). Statins are 3-hydroxy-3-methylglutaryl coenzyme A (HMG-CoA) reductase inhibitors with the primary function of reducing endogenous LDL cholesterol. Some previous studies found that statins exert fascinating pleiotropic effects in addition to reducing LDL cholesterol, such as anti-inflammatory, antithrombotic, and antioxidant effects (5), which can improve vascular function and improve ventricular remodeling (6). There is evidence that statins can reduce the risk of various cardiovascular events in patients with diabetes (7) resulting in statins becoming the first choice of lipid-lowering drugs for reducing CVD risk. Type 2 diabetes leads to an increase in the prevalence of lipid abnormalities, which increases the risk of CVD. Therefore, current guidelines generally recommend the use of moderate or high-intensity statins in patients with type 2 diabetes (8, 9). However, there is still controversy about whether statins are important in acute myocardial infarction (AMI) patients with diabetes, and there are still few studies. Some retrospective registration studies showed that the statin group showed lower major adverse cardiac events, all-cause mortality, cardiac death than the non-statin group (10, 11). However, studies have shown that the beneficial effect of statins in AMI patients with diabetes has not been confirmed (12). Most previous studies have focused exclusively on the protective effect of statins on cardiovascular events, and so the overall risk–benefit balance of statins for patients with AMI and diabetes needs to be reassessed. Compared with other types of lipid-lowering drugs, the advantage of statins for the prognosis of patients with AMI has yet to be determined. We therefore hypothesized that patients with AMI and diabetes who receive statins have lower intensive care unit (ICU) and in-hospital mortality rates than those who do not receive lipid-lowering drugs. We tested this hypothesis using the Medical Information Mart Intensive Care-IV (MIMIC-IV) database.

## Methods

### Data source and population

**Figure 1:**
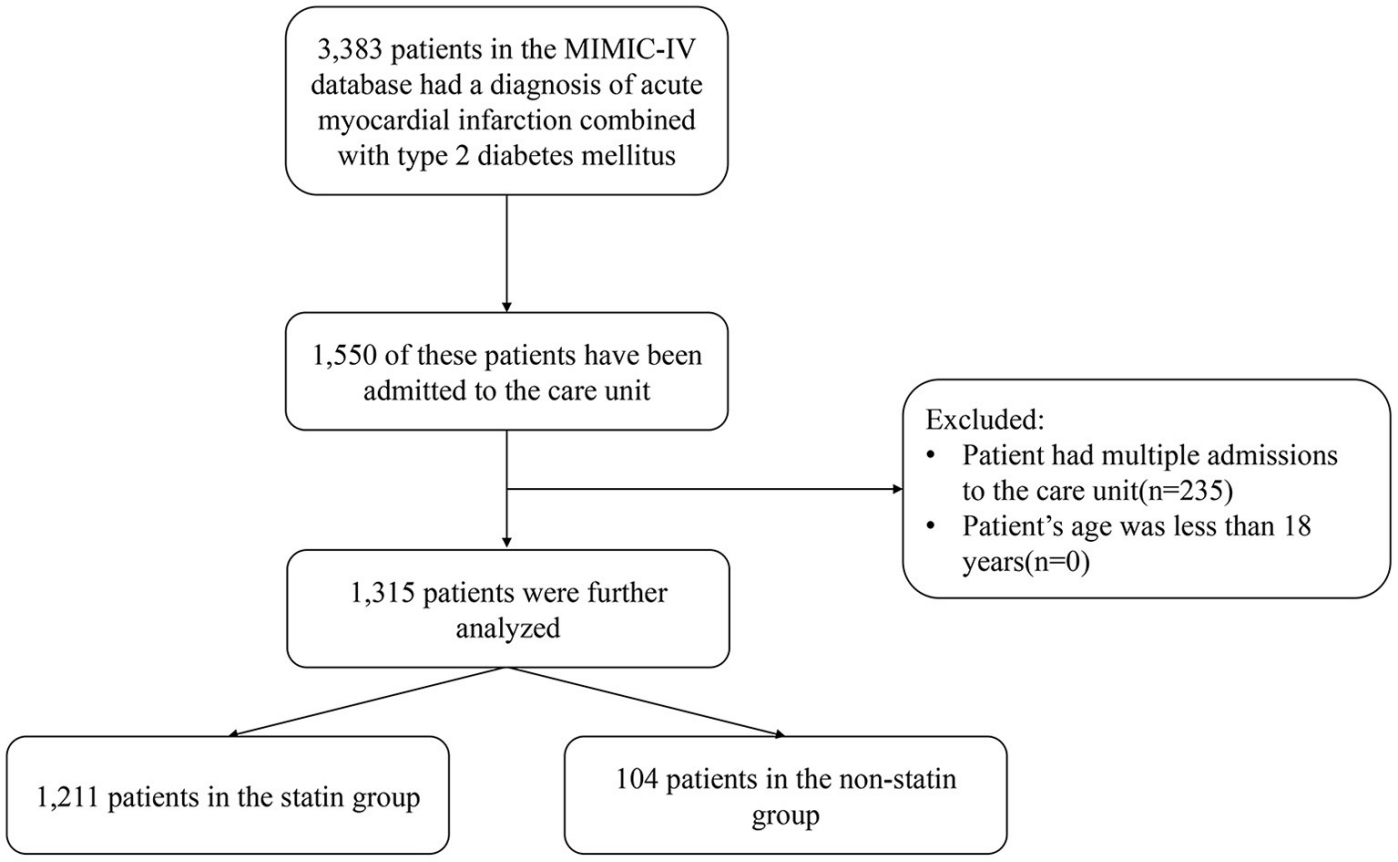
Flow chart for study participants.

This was a retrospective study based on version 1.0 of the MIMIC-IV database, which a vertical, single-center database that includes all patients admitted to the Beth Israel Deaconess Medical Center (BIDMC) emergency department or ICU during 2008–2019 (13). We obtained access to the database after completing the recognized “Protecting Human Research Participants” course. The institutional review boards of BIDMC and MIT approved any researcher meeting the data user requirements to use the MIMIC-IV database, and exempted them from the requirement to obtained informed consent from patients. This study included all patients with AMI complicated with diabetes in the database, and excluded patients younger than 18 years. We only extracted the information of patients hospitalized and admitted to ICU for the first time, and excluded those with multiple hospitalization records (Figure 1).

### Data extraction

Structured Query Language was used to extract the following information from the database: age, gender, weight, ethnicity, acute physiology score-III (APSIII), first care unit, ventilator and vasopressor use, continuous renal replacement therapy (CRRT), percutaneous coronary intervention (PCI), and coronary artery bypass grafting (CABG) (14). Major comorbidities included diabetes, hyperlipidemia, hypertension, congestive heart failure, peripheral vascular disease, cerebrovascular disease, chronic pulmonary disease, renal disease, malignant cancer, and liver disease. The average values of the following vital signs were collected: mean blood pressure (MBP), heart rate, respiratory rate, temperature, and peripheral capillary oxygen saturation (SpO_2_) within 24 h of ICU admission. The following first laboratory test results in the ICU were collected: white blood cell (WBC), hemoglobin, platelet, red cell distribution width (RDW), anion gap, potassium, calcium total, creatinine, urea nitrogen, glucose, international normalized ratio (INR), urine output, total bilirubin, lactate, and the peak myocardial infarction markers of troponin T and creatine kinase isoenzyme (CKMB). The end point of this study was whether patients died in hospital, and the secondary outcome was ICU mortality.

### Statistical analyses

We first used the multiple imputation method to supplement variables with <20% missing data using the R software “mice” package. The patients in this study were divided into statin and non-statin groups according to whether they had been treated with an antihyperlipidemic agent (HMG-CoA reductase inhibitor). After the data cohort was determined, all categorical variables were expressed in numbers and percentages, and chi-square and Fisher's exact tests were used to determine the differences between the two groups. All continuous variables were expressed as median and interquartile range values, and differences between the two groups was determined using the Mann-Whitney U test. Kaplan-Meier and Cox proportional-hazards regression models were applied to analyze the correlation between the two groups and the outcomes. Log-rank tests were performed as non-parametric analyses to compare the survival distributions of the two groups. Two Cox models were constructed: model 1 had no adjustments, and model 2 was adjusted for all of the above-mentioned covariates. All statistical analyses were performed using R software (version 4.0.1), and *P* < 0.05 (two-sided) was considered indicative of statistical significance.

## Results

### Baseline characteristics

We finally included and analyzed 1,315 patients with AMI and diabetes from the MIMIC-IV database, among which 1,211 patients used statins during hospitalization (statins group) and 104 did not (non-statin group). The baseline data of the two groups are listed in Table 1. The overall in-hospital mortality of patients with diabetes complicated with AMI was 17.2%, and the total ICU mortality rate was 12.6%. The in-hospital mortality rate was significantly lower in the statin than in the non-statin group (13.9 and 55.8%, respectively; *P* < 0.001), as was the ICU mortality rate (9.3 and 51.0%, respectively; *P* < 0.001). In the statin group, the proportions of those who received CRRT and vasoactive drugs were lower (1.8 and 5.8%, respectively; *P* = 0.02), and the proportions of those that received PCI (26.3 and 12.5%, respectively; *P* = 0.003) and CABG (29.4 and 4.8%, respectively; *P* < 0.001) were higher. There were more patients with hyperlipidemia in the statin than the non-statin group (66.6 and 45.2%, respectively; *P* < 0.001).

**Table 1:** The baseline data of the statins group and non-statin group.

	**Statin group**	**Non-statin group**	***p***
	***n =* 1,211**	***n =* 104**	
Age (year)	71.00 (63.00, 79.00)	75.00 (63.75, 83.00)	0.064
Gender (%)			0.01
Male	766 (63.3)	52 (50.0)	
Female	445 (36.7)	52 (50.0)	
Ethnicity (%)			0.908
White	712 (58.8)	60 (57.7)	
Others	499 (41.2)	44 (42.3)	
Weight (kg)	84.82 (71.26, 99.12)	78.03 (69.16, 93.82)	0.025
APSIII	44.00 (34.00, 61.00)	67.00 (49.00, 93.75)	<0.001
First care unit (%)			<0.001
CCU	923 (76.2)	45 (43.3)	
others	288 (23.8)	59 (56.7)	
Vasopressor (%)			<0.001
No	855 (70.6)	46 (44.2)	
Yes	356 (29.4)	58 (55.8)	
Ventilator (%)			0.109
No	571 (47.2)	40 (38.5)	
Yes	640 (52.8)	64 (61.5)	
CRRT (%)			0.02
No	1,189 (98.2)	98 (94.2)	
Yes	22 (1.8)	6 (5.8)	
PCI (%)			0.003
No	893 (73.7)	91 (87.5)	
Yes	318 (26.3)	13 (12.5)	
CABG (%)			<0.001
No	855 (70.6)	99 (95.2)	
Yes	356 (29.4)	5 (4.8)	
**Comorbidities**			
Diabetes complicated (%)			0.632
No	698 (57.6)	63 (60.6)	
Yes	513 (42.4)	41 (39.4)	
Hyperlipidemia (%)			<0.001
No	404 (33.4)	57 (54.8)	
Yes	807 (66.6)	47 (45.2)	
Hypertension (%)			0.507
No	722 (59.6)	66 (63.5)	
Yes	489 (40.4)	38 (36.5)	
Congestive heart failure (%)			0.874
No	508 (41.9)	45 (43.3)	
Yes	703 (58.1)	59 (56.7)	
Peripheral vascular disease (%)			1
No	1,021 (84.3)	88 (84.6)	
Yes	190 (15.7)	16 (15.4)	
Cerebrovascular disease (%)			0.095
No	1,015 (83.8)	80 (76.9)	
Yes	196 (16.2)	24 (23.1)	
Chronic pulmonary disease (%)			0.627
No	923 (76.2)	82 (78.8)	
Yes	288 (23.8)	22 (21.2)	
Renal disease (%)			0.841
No	694 (57.3)	58 (55.8)	
Yes	517 (42.7)	46 (44.2)	
Liver disease (%)			<0.001
No	1,127 (93.1)	85 (81.7)	
Yes	84 (6.9)	19 (18.3)	
Malignant cancer (%)			0.07
No	1,134 (93.6)	92 (88.5)	
Yes	77 (6.4)	12 (11.5)	
**Vital signs**			
MBP (mmHg)	75.48 (69.88, 82.59)	71.87 (64.26, 79.29)	<0.001
Heart rate (bpm)	81.08 (72.04, 90.19)	87.00 (78.01, 100.84)	<0.001
Respiratory rate (insp/min)	18.85 (16.88, 21.00)	20.73 (17.59, 23.68)	<0.001
Temperature (°C)	36.77 (36.60, 36.94)	36.68 (36.50, 37.02)	0.109
SpO_2_ (%)	97.09 (95.84, 98.27)	96.59 (95.24, 98.61)	0.242
**Laboratory tests**			
Troponin T (ng/ml)	1.21 (0.31, 3.47)	0.65 (0.16, 2.43)	0.036
CKMB (ng/ml)	11.00 (4.00, 36.50)	14.50 (4.00, 51.25)	0.367
WBC (k/ul)	9.60 (7.50, 13.10)	11.40 (7.50, 17.70)	0.013
Hemoglobin (g/dl)	11.20 (9.50, 12.80)	10.00 (7.97, 11.70)	<0.001
Platelet (k/ul)	203.00 (158.00, 255.75)	182.50 (126.25, 229.75)	0.003
RDW (%)	14.10 (13.20, 15.50)	15.60 (14.10, 17.10)	<0.001
Anion Gap (mEq/l)	16.00 (13.00, 19.00)	19.00 (16.00, 23.50)	<0.001
Lactate (mmol/l)	1.60 (1.20, 2.30)	3.65 (2.28, 8.12)	<0.001
Potassium (mEq/l)	4.20 (3.90, 4.60)	4.60 (4.05, 5.20)	<0.001
Calcium Total (mg/dL)	8.70 (8.20, 9.10)	8.20 (7.70, 8.90)	<0.001
Glucose (mg/dl)	174.00 (130.00, 237.50)	195.00 (124.50, 315.00)	0.075
INR	1.20 (1.10, 1.30)	1.55 (1.20, 2.30)	<0.001
Creatinine (md/dl)	1.20 (0.90, 1.90)	1.90 (1.17, 2.90)	<0.001
Urea Nitrogen (mg/dl)	25.00 (17.00, 40.00)	37.50 (23.00, 56.25)	<0.001
Urine output (ml)	1535.00 (940.00, 2225.00)	785.00 (231.50, 1448.00)	<0.001
Bilirubin Total (mg/dl)	0.50 (0.30, 0.80)	0.80 (0.50, 1.50)	<0.001
**ICU mortality (%)**			
No	1,098 (90.7)	51 (49.0)	<0.001
Yes	113 (9.3)	53 (51.0)	
**In-hospital mortality (%)**			
No	1,043 (86.1)	46 (44.2)	<0.001
Yes	168 (13.9)	58 (55.8)	

APSIII, acute physiology score-III; CCU, cardiac care unit; CRRT, continuous renal replacement therapy; PCI, percutaneous coronary intervention; CABG, coronary artery bypass grafting; MBP, mean blood pressure; SpO_2_, peripheral capillary oxygen saturation; CKMB, creatine kinase isoenzyme; WBC, white blood cell; RDW, red cell distribution width; INR, international normalized ratio.

### Clinical outcomes

**Figure 2:**
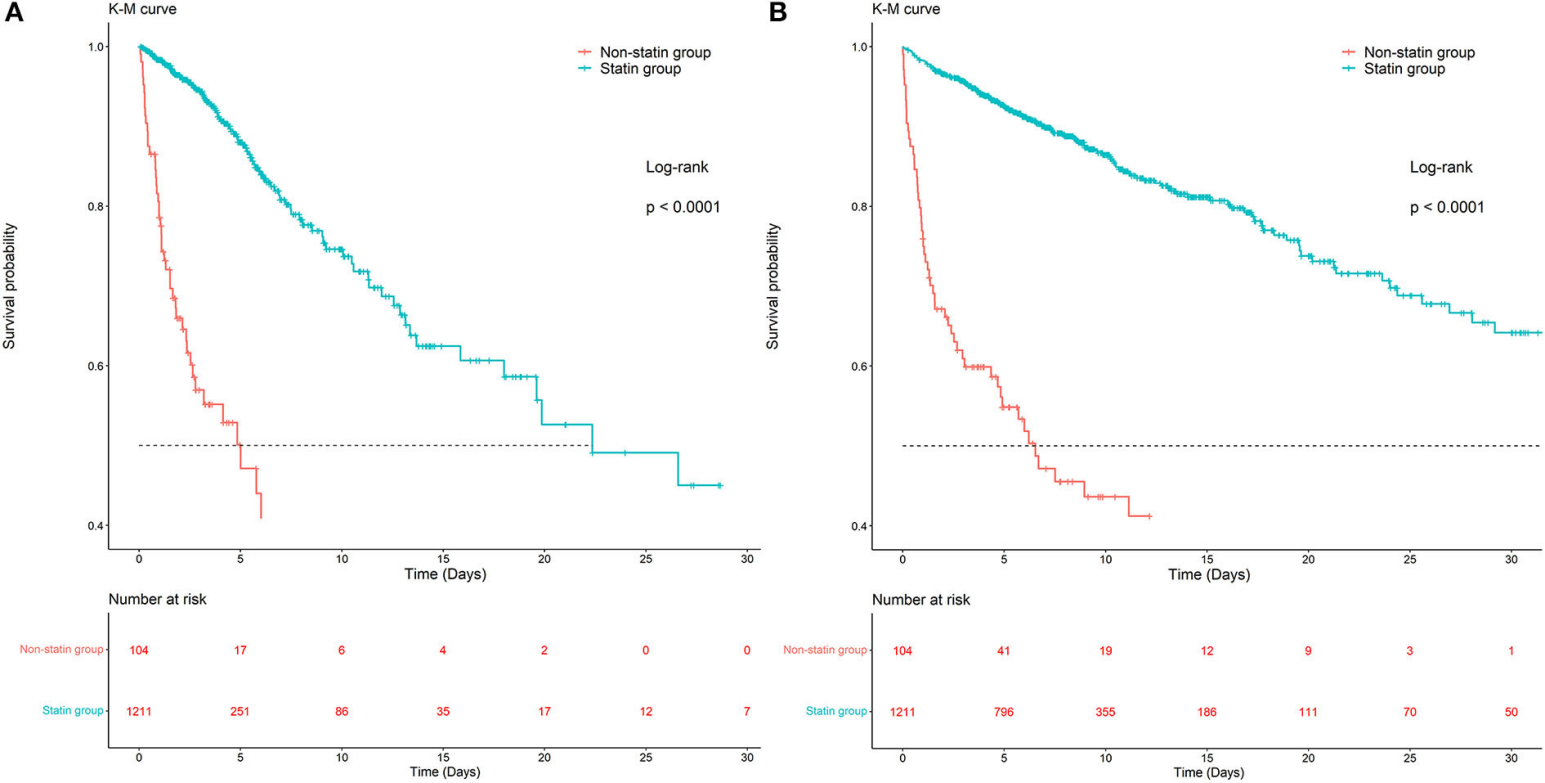
Kaplan-Meier survival curves of ICU and in-hospital mortality in the statin group and non-statin group. **(A)** Kaplan-Meier survival curves of ICU mortality in the statin group and non-statin group. **(B)** Kaplan-Meier survival curves of in-hospital mortality in the statin group and non-statin group.

Kaplan-Meier survival curves demonstrated that the survival probability was significantly higher in the statin group than in the non-statin group (*p* < 0.0001, Figure 2). Two Cox models were constructed: model 1 had no adjustments, and in model 2 we adjusted for age, gender, weight, ethnicity, APSIII, ventilator use, vasopressor use, CRRT use, PCI use, CABG use, diabetes, hyperlipidemia, hypertension, congestive heart failure, peripheral vascular disease, cerebrovascular disease, chronic pulmonary disease, renal disease, malignant cancer, liver disease, MBP, heart rate, respiratory rate, temperature, SpO_2_, WBC, hemoglobin, platelet, RDW, anion gap, potassium, calcium total, creatinine, urea nitrogen, glucose, INR, urine output, total bilirubin, lactate, troponin T and CKMB. After adjusting for all of the above-mentioned covariates using Cox proportional-hazards models, the risks of ICU and in-hospital mortality were significantly lower in the statin than the non-statin group, with HRs of 0.14 (95% CI = 0.08–0.27, Table 2) and 0.28 (95% CI = 0.17–0.47, Table 2), respectively.

**Table 2:** Clinical outcomes between statin group and non-statin group.

	**Non-statin group**	**Statin group**	
	**HR (95%CI)**	**HR (95%CI)**	***p*** **-value**
**ICU Mortality**
Unadjusted	Reference	0.16 (0.12,0.22)	<0.001
Adjusted	Reference	0.14 (0.08,0.27)	<0.001
**In-hospital Mortality**
Unadjusted	Reference	0.17 (0.13,0.24)	<0.001
Adjusted	Reference	0.28 (0.17,0.47)	<0.001

HR, Hazard Ratio; ICU, intensive care unit.

### Subgroup analyses

Statins are most commonly used to reduce LDL cholesterol, and so a subgroup analysis was applied to the effect of statins on clinical outcomes in subgroups with hyperlipidemia. There were 854 patients with and 461 without hyperlipidemia. In the cohort with hyperlipidemia, the risks of ICU and in-hospital death were lower in the statin group than in the non-statin group, with HRs of 0.07 (95% CI = 0.02–0.20, Table 3) and 0.16 (95% CI = 0.07–0.41, Table 3), respectively; the corresponding values in the cohort without hyperlipidemia were 0.12 (95% CI = 0.04–0.40, Table 3) and 0.36 (95% CI = 0.16–0.84, Table 3), respectively.

**Table 3:** The effect of statins on clinical outcomes in subgroups with hyperlipidemia.

	**ICU mortality**			**In-hospital mortality**		
	**HR (95%CI)**	***p*-value**	**p-interaction**	**HR (95%CI)**	***p*-value**	**p-interaction**
Hyperlipidemia			0.595			0.086
No (*n =* 461)	0.12 (0.04,0.40)	0.001		0.36 (0.16,0.84)	0.018	
Yes (*n =* 854)	0.07 (0.02,0.20)	<0.001		0.16 (0.07,0.41)	<0.001	

## Discussion

By collecting the statin use data of hospitalized patients with AMI complicated with diabetes, and comparing them with patients who did not use statins or other lipid-lowering drugs, this retrospective study found that statins had significant clinical benefits on the prognosis of hospitalized patients with diabetes and AMI. Compared with non-statins and other types of lipid-lowering drugs, statins can significantly reduce ICU and in-hospital mortality rates in patients with AMI and diabetes. In the population without hyperlipidemia, statins can still reduce the mortality in patients with AMI and diabetes. Statins can reduce serum LDL cholesterol levels. Current guidelines strongly recommend administering statins at high concentrations or at the maximum tolerance level of patients with AMI without contraindications (15, 16). Some previous studies have demonstrated that the benefits of statins far outweigh their potential risks (17, 18). Statin use is related to difficulty in controlling blood glucose in diabetes and pre-diabetes, but they greatly reduce the risk of cardiovascular events (19). In the current study, patients who took statins had significantly lower ICU mortality and in-hospital mortality risks than those who did not, with HRs of 0.16 (95% CI = 0.12–0.22) and 0.17 (95% CI = 0.13–0.24), respectively. After adjusting for some possible confounders, the advantage of statins in reducing the risk of death remained. In our study, adjusted ICU and in-hospital mortality rates were also significantly reduced, with HRs of 0.14 (95% CI = 0.08–0.27) and 0.28 (95% CI = 0.17–0.47), respectively.

While the present patients in the non-statin group did not use statins to control blood lipids, they may have used other types of lipid-lowering drugs such as fibrates, ezetimibe, and niacin. Several past meta-analyses have found that although fibrates can reduce the risk of cardiovascular events (20–22), they will not reduce all-cause or CVD mortality. Similarly, a meta-analysis found that a combination therapy of statins and fibrates had no more clinical benefits than statins alone (23). A previous study also found no difference in cardiac or all-cause or myocardial infarction mortality between simvastatin-ezetimibe and high-intensity statins in a population with AMI, although a significant reduction in the repeated revascularization rate was observed (24). A previous meta-analysis compared the effects of statins, ezetimibe, and PCSK9 inhibitors, and found that statins had the greatest probability of reducing all-cause and cardiovascular mortality (25). In another study on atherosclerotic vascular disease, compared with statins alone, the combination of niacin-laropiprant and statins not only failed to reduce cardiovascular event risk, but also increased the risks of bleeding, infection, and new-onset diabetes (26). These findings consistently suggest that statins have more benefits than other types of lipid-lowering drugs in patients at higher risks of cardiovascular events, and statins remain the most effective way to reduce mortality from these events.

Some novel conclusions can be drawn from this retrospective cohort study. Since statins are the most commonly used treatment for blood lipid control, we performed a subgroup analysis of whether patients were complicated with hyperlipidemia. In patients with hyperlipidemia, statins could significantly reduce ICU and in-hospital death risks, with HRs of 0.07 (95% CI = 0.02–0.20, *P* < 0.001) and 0.16 (95% CI = 0.07–0.41, *P* < 0.001), which is consistent with many guidelines (15, 16). The current study also demonstrated that statins can reduce ICU and in-hospital mortality rates in patients without hyperlipidemia, with HRs of 0.12 (95% CI = 0.04–0.40, *P* = 0.001) and 0.36 (95% CI = 0.16–0.84, *P* = 0.018), respectively. This suggests that statins act via other mechanisms to improve the prognosis of patients with AMI and diabetes. Some previous studies have found that in addition to reducing LDL cholesterol, statins also exert fascinating pleiotropic effects, including anti-inflammatory, inhibiting oxidative stress, antiplatelet aggregation, antithrombosis, and improving vascular tension (27). These effects are essential to inhibiting atherosclerotic plaque progression and thus contribute to an overall reduction of the CVD death risk. However, the exact underlying molecular mechanism has not been determined, and so further research is still needed to clarify it.

Our study had some limitations. First, this study is a single center regression study, which questions the universality of conclusion. Secondly, this study lacked data related to new-onset diabetes, such as fasting blood glucose and glycosylated hemoglobin before and after statins, so it was not able to explain the direct relationship between statins and new-onset diabetes. Third, LDL cholesterol is very important for the population of this study, but due to the limitations of the database, we failed to obtain these data. Finally, because most patients in the statin group in this study were treated with atorvastatin, we cannot provide the results of different statins separately. Notwithstanding these limitations, this study demonstrated that statins have protective effects on patients with AMI and diabetes.

## Conclusions

Compared with non-statins and other types of lipid-lowering drugs, statins can significantly reduce ICU and in-hospital mortality rates in patients with AMI and diabetes. Even in the population without hyperlipidemia, statins can still reduce the mortality in patients with AMI and diabetes. Although prospective randomized trials are needed to confirm the current results, they strongly suggest that statins have a protective effect on patients with AMI and diabetes.

## Data availability statement

Publicly available datasets were analyzed in this study. This data can be found here: The data were available on the MIMIC-IV website at https://mimic.physionet.org/, https://doi.org/10.13026/a3wn-hq05.

## Author contributions

XL and LZ created the study protocol, performed the statistical analyses, and wrote the first manuscript draft. SL conceived the study and critically revised the manuscript. DH assisted with data collection and manuscript editing. TH and HL assisted the analysis and explain of statistical methods. HY assisted with manuscript revision and data confirmation. JL contributed to data interpretation and manuscript revision. All authors read and approved the final manuscript.

## Funding

This study received financial support from the National Natural Science Foundation of China (Nos. 82072232 and 81871585), the Natural Science Foundation of Guangdong Province (No. 2018A030313058), Technology and Innovation Commission of Guangzhou Science, China (No. 201804010308), Guangdong Provincial Key Laboratory of Traditional Chinese Medicine Informatization (2021B1212040007).

## Conflict of interest

The authors declare that the research was conducted in the absence of any commercial or financial relationships that could be construed as a potential conflict of interest.

## Publisher's note

All claims expressed in this article are solely those of the authors and do not necessarily represent those of their affiliated organizations, or those of the publisher, the editors and the reviewers. Any product that may be evaluated in this article, or claim that may be made by its manufacturer, is not guaranteed or endorsed by the publisher.
